# *N*^6^-Methyladenosine (m^6^A)-circHECA Recruiting FUS Promotes Differentiation of SHF Stem Cells into Hair Follicle Lineages Through Stabilizing FOXM1 mRNA to Activate NOTCH Pathway in Cashmere Goats

**DOI:** 10.3390/ani16162625

**Published:** 2026-08-21

**Authors:** Xinjiang Zhang, Yubo Zhu, Jincheng Shen, Ruqing Xu, Man Bai, Yixing Fan, Taiyu Hui, Qi Zhang, Wenlin Bai

**Affiliations:** 1 College of Animal Science & Veterinary Medicine, Shenyang Agricultural University, Shenyang 110866, China; 2 Engineering Research Center for Animal Molecular Genetics and Breeding of Liaoning Province, Shenyang 110866, China

**Keywords:** circHECA, *N*^6^-methyladenosine, SHF stem cells, induced differentiation, hair follicle lineage, cashmere goats

## Abstract

Cashmere goats are widely distributed in the cold, arid and semi-arid remote regions of northern China. Their main economic value is the production of precious cashmere fibers. The differentiation of second hair follicle (SHF) stem cells into hair follicle lineages plays a crucial role in SHF regeneration as well as in the morphogenesis and growth of cashmere fibers; however, its precise molecular mechanism is still unclear. In this study, we found that *N*^6^-methyladenosine (m^6^A)-circHECA recruiting FUS promoted the differentiation of SHF stem cells into hair follicle lineages through stabilizing FOXM1 mRNA in SHF stem cells, thereby activating the NOTCH pathway in cashmere goats. Furthermore, we confirmed that the m^6^A modification of circHECA is required for the FUS/FOXM1-mediated NOTCH signaling pathway to facilitate the differentiation of SHF stem cells into hair follicle lineages via transfecting circHECA m^6^A-deficient mutants. Our results contribute to elucidating the functional mechanism of m^6^A-circHECA in the differentiation of SHF stem cells into hair follicle lineages in cashmere goats.

## Simple Summary

Cashmere goats are widely distributed in the cold, arid and semi-arid remote regions of northern China. Their main economic value is the production of precious cashmere fibers. In the process of cashmere production, the differentiation of second hair follicle (SHF) stem cells into hair follicle lineages plays a crucial role, which is closely related to SHF regeneration as well as the morphogenesis and growth of cashmere fibers; however, its precise molecular mechanism is still unclear. In this study, we found that *N*^6^-methyladenosine (m^6^A)-circHECA recruiting FUS promoted the differentiation of SHF stem cells into hair follicle lineages through stabilizing FOXM1 mRNA to activate the NOTCH pathway in cashmere goats. These findings provide a potential target for artificially regulating the differentiation of SHF stem cells into hair follicle lineages in cashmere goats.

## 1. Introduction

Cashmere goats, a unique type of goat, are widely distributed in the cold, arid or semi-arid remote regions of northern China [1]. Their main economic value is provided through the production of precious cashmere fibers. As a rare and high-quality natural protein fiber, the textiles of cashmere fibers are highly preferred and praised by consumers due to their typical features such as soft handle, lightness, comfort, luster, and warmth retention [2]. Cashmere fibers are derived from the secondary hair follicles (SHFs) of cashmere goats. In the production process of cashmere, the differentiation of SHF stem cells into hair follicle lineages under the induction from dermal papilla cell (DPC)-derived signals plays a crucial role, which is closely related to SHF regeneration, morphogenesis, and growth of cashmere fibers [3].

The differentiation of hair follicle stem cells into hair follicle lineages is an extremely complex biological process that is precisely co-regulated by a variety of endogenous regulatory factors at multiple levels. In recent years, non-coding RNAs were essentially implicated in the differentiation of hair follicle stem cells into hair follicle lineages including miRNAs, lncRNAs and circRNAs. There is evidence that miR-125b [4] and miR-22 can negatively regulate the differentiation process of hair follicle stem cells [5]. lncRNA AK015322 was reported to promote the differentiation of hair follicle stem cells into hair follicle lineages through the PI3K-AKT signaling pathway [6]. Also, some circRNAs were demonstrated to facilitate the differentiation of SHF stem cells into hair follicle lineages via miRNA-mediated mechanisms, such as circRNA-1926 [7], circRNA-0100 [8], and circRNA-1967 [9].

Interestingly, extensive *N*^6^-Methyladenosine modification (m^6^A) sites have been revealed in some circRNA molecules, with essential functional roles [10,11]. It was reported that m^6^A modifications of circRNAs might be widely involved in the formation of pork quality [12]. Also, m^6^A modification was implicated in the regulation of circRNA-08436 in lipid metabolism in goat mammary glands [13]. More recently, the m^6^A modification of circCDK14 was proven to promote its nuclear export, which is subjected to the co-regulation of SRSF3 and hnRNPA1, thereby enhancing the functional role of circCDK14 in intramuscular fat deposition of yaks [14]. Some m^6^A-modified circRNAs have also been characterized from SHFs or skin tissues in cashmere goats [15,16,17]. Furthermore, it has been demonstrated that circRNA-ZNF638 and circERCC6 positively regulate the induced activation of SHF stem cells via m^6^A-mediated miR-412-3p/BNC2 and miR-361-5p/Wnt5a axes in cashmere goats, respectively [18,19].

m^6^A-modified circHECA (m^6^A-circHECA) was previously characterized in cashmere goat SHFs, along with the mechanism regulating its expression and potential regulatory network [20]. More recently, a study implicated m^6^A-circHECA in fate of SHF stem cells through miRNA-mediated pathway in cashmere goats [21]. However, an increasing number of studies indicate that circRNAs can also exert their functional roles in biological cells via interacting with RNA binding proteins [22,23]. In this study, therefore, we mainly investigated the functional significance of m^6^A-circHECA in the differentiation of SHF stem cells into hair follicle lineages in cashmere goats, with an exploration of the mechanism mediated by its potential RNA binding proteins. Our results contribute to elucidating the functional mechanism of m^6^A-circHECA in the differentiation of SHF stem cells into hair follicle lineages in cashmere goats.

## 2. Materials and Methods

### 2.1. RNA Samples and Co-Culture of SHF Stem Cells with Dermal Papilla Cells

Here, for the analysis of m^6^A-circHECA expression in SHF bulge, total RNA was used, which was isolated from the SHF bulge of cashmere goats in our recent investigation [19]. For the expression correlation of FUS and FOXM1 mRNAs in SHFs, the total RNA was utilized, which was isolated from cashmere goat SHFs in our other study [20]. The SHF stem cells were co-cultured with dermal papilla cells for inducing differentiation into hair follicle lineages; the two types of cells have been stored in our laboratory [19]. The co-culture was performed in a transwell device, as described in a previous publication [3]. In brief, after plating SHF stem cells (passage 3) in six-well plate at a density of 1 × 10^4^ cells/mL, a transwell insert was added, with the dermal papilla cells (passage 3) being seeded at a density of 1 × 10^4^ cells/mL for inducing the differentiation of SHF stem cells. The co-culture of the two types of cells (SHF stem cells and dermal papilla cells) was carried out in fresh DMEM/F12 medium (Hyclone, Logan, UT, USA) at 37 °C under 5% CO_2_. The medium was changed every 2 days. After co-culturing for 7 days [3], the SHF stem cells were harvested, and total RNA and protein were isolated with a RNAiso reagent kit (TaKaRa, Dalian, China) and a Protein Extraction Kit (Solarbio, Beijing, China), respectively. As for the actinomycin D treatment, the SHF stem cells were treated with actinomycin D for 4 h and 8 h [24]. Subsequently, total RNA was isolated, and the RNA samples were analyzed by an RT-qPCR technique.

### 2.2. Analysis of Overexpression and Knockdown of circHECA in SHF Stem Cells

We used pcDNA3.1 (+) circRNA mini-vector (Addgene, Cambridge, MA, USA) for constructing the overexpression vectors of circHECA (or its corresponding mutants) in SHF stem cells. The corresponding mutants of circHECA were created with a QuikChange Lightning Multi Site-Directed Mutagenesis Kit (Agilent Technologies, Santa Clara, CA, USA). The overexpression vectors of circHECA (or its corresponding mutants) were transfected into SHF stem cells using a Lipofectamine 3000 (Invitrogen, Carlsbad, CA, USA) according the manufacturer’s recommendations. Three siRNAs were used for circHECA knockdown in SHF stem cells, including si-circHECA-1^#^ (5′-GCACAAGGGGC- TCTTGAAGGTG-3′), si-circHECA-2^#^ (5′-AAGGGGCTCTTGAAGGTGACAG-3′), and si-circHECA-3^#^ (5′-GTTGCACAAGGGGCTCTTGAAG-3′). The three siRNAs were previously designed in our laboratory [21]. Lipofectamine RNAiMAX kits (Invitrogen, Shanghai, China) were utilized for transfecting the siRNAs into SHF stem cells following the manufacturer’s recommendations.

### 2.3. Overexpression and/or Knockdown of FUS and FOXM1 in SHF Stem Cells

For the overexpression of FUS and FOXM1 genes in SHF stem cells of cashmere goats, PCR amplifications were performed to obtain the coding sequence of goat FUS and FOXM1 mRNAs using Phanta Max Super-Fidelity DNA Polymerase (Vazyme, Nanjing, China), respectively. The amplified products of FUS and FOXM1 mRNAs were purified and cloned into pcDNA3.1(+) vectors. The generated overexpression vectors of FUS (pcDNA3.1-FUS) and FOXM1 (pcDNA3.1-FOXM1) were transfected into SHF stem cells of cashmere goats using Lipofectamine 3000 according to the manufacturer’s recommendations (Invitrogen, Carlsbad, CA, USA). For the knockdown of FUS mRNA in SHF stem cells, three small interference RNAs (siRNA-FUS-1^#^, siRNA-FUS-2^#^, and siRNA-FUS-3^#^) targeting FUS mRNAs were commercially obtained from Shanghai GenePharma. Co., Ltd. with its negative control (si-control) (GenePharma, Shanghai, China). The small RNAs were transfected into SHF stem cells with Lipofectamine RNAiMAX kits (Invitrogen, Shanghai, China) following the manufacturer’s recommendations.

### 2.4. RNA Immunoprecipitation (RIP) and RNase R Treatment

The RNA immunoprecipitation (RIP) assay was carried out by a EZ-Magna RIP RNA Binding Protein Immunoprecipitation Kit (Millipore, Burlington, MA, USA) according to the manufacturer’s guide. The anti-IgG, anti-FUS, and anti-EIF4B antibodies were commercially obtained from Abcam (Cambridge, UK) and anti-RBMX from Cell Signaling Technology (Boston, MA, USA), respectively. The SHF stem cells were firstly fixed using 1% formaldehyde and then were lysed with lysis buffer. Cell lysates were incubated with specific antibodies at 4 °C overnight, including anti-IgG (ab172730, Abcam), anti-FUS (ab243880, Abcam), anti-EIF4B (ab245618, Abcam), and anti-RBMX (14794, Cell Signaling). Then, the lysates were incubated with protein A/G magnetic beads for 2 h (Bimake, Beijing, China). After washing, the immunoprecipitated RNAs were purified, and the enrichment of circHECA and FOXM1 mRNA was detected using RT-qPCR. As for the RNase R treatment, the total RNA from SHF stem cells was treated with RNase R for 30 min. Subsequently, the RNA samples were analyzed by RT-qPCR.

### 2.5. Primer Designing and Quantitative Real-Time PCR Reactions (RT-qPCR)

For detecting the expression of m^6^A-circHECA, the divergent primer pair that was designed in a previous investigation was used [20]. The corresponding convergent primer pairs were also cited from previous publications for the mRNA analysis of GAPDH [18], KRT8 [7], KRT17 [7], and FOXM1 [25]. The rest of the convergent primer pairs of the implicated genes were designed with Primer Premier 5.0 software (http://www.premierbiosoft.com, accessed on 9 March 2025). All the designed primer pairs yielded a single dissociation peak with amplification efficiencies of 90–110%, which ensured reliable quantification. All primers used in this study are listed in Table 1.

The first-strand cDNA was synthesized by an M-MuLV cDNA synthesis kit with random primers (Sangon, Shanghai, China). We performed the RT-qPCR assay with a light Cycler 480 real-time PCR system (Roche Diagnostics, Mannheim, Germany). The RT-qPCR reactions were conducted in a final volume of 25 µL with TB Green Premix Ex Taq II (Tli RNaseH Plus; TaKaRa, Dalian, China) following the manufacturer’s guide. The reaction assay consisted of 1.0 µL (10 µM) of each primer, 2.0 µL of first-strand cDNA solution, 12.5 µL of TB Green Premix Ex Taq II (TliRNaseH Plus; TaKaRa, Dalian, China), and 8.5 µL of PCR-grade ddH_2_O water. The thermal cycling parameters were set as: a single cycle (95 °C for 30 s), followed by 40 cycles: 95 °C for 5 s, 53 °C to 58 °C (Table 1) for 30 s, and 72 °C for 30 s. Ultimately, the relative expression abundance of each analyzed gene was measured by the 2^−∆∆Ct^ method [26], where the housekeeping gene GAPDH was amplified as the internal reference.

### 2.6. Statistical Data Analysis

Each experiment was performed in at least three biological replicates. The obtained data are presented as mean ± standard deviation and were analyzed using SPSS 17.0 software (SPSS Inc., Chicago, IL, USA). Differences between two groups were compared with Student’s *t*-test, and differences among three or more groups were compared with one-way analysis of variance (ANOVA), followed by Dunnett’s test for multiple comparisons. A *p*-value less than 0.05 was considered the threshold of statistical significance. Ultimately, the statistical results were displayed by GraphPad Prism for Windows (Version 8.3.0, San Diego, CA, USA).

## 3. Results and Discussion

### 3.1. Characterization of circHECA and circHECA Overexpression Promotes Differentiation of SHF Stem Cells into Hair Follicle Lineages in Cashmere Goats

**Figure 1:**
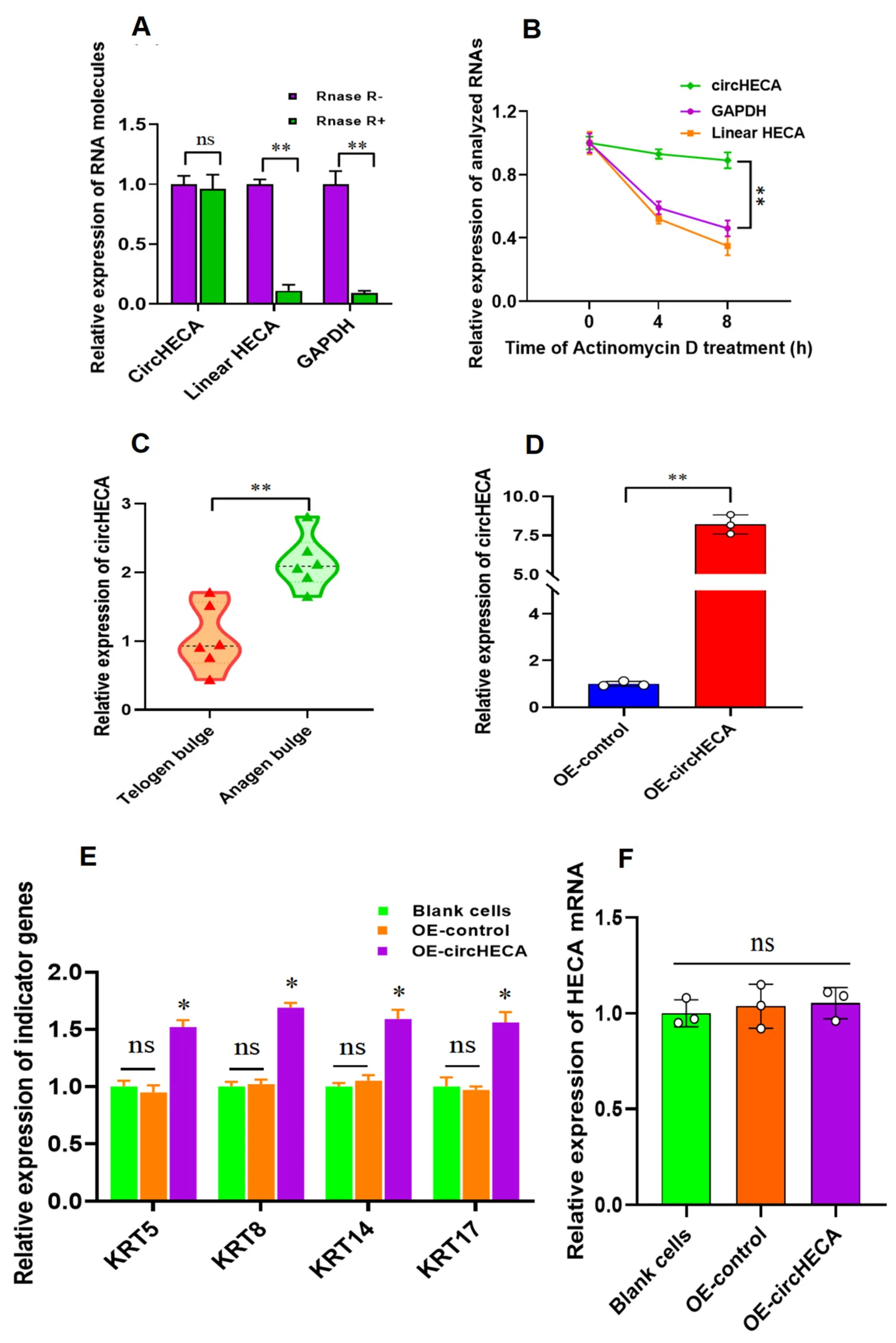
Characterization of circHECA. circHECA overexpression promotes the differentiation of SHF stem cells into hair follicle lineages in cashmere goats. (**A**) After RNase R treatment, the stability of circHECA was detected through RT-qPCR analysis through comparison with that of linear HECA and GAPDH mRNA. (**B**) Stability of circHECA was evaluated in SHF stem cells of cashmere goats through RT-qPCR analysis through comparison with that of linear HECA and GAPDH mRNA upon treatment with actinomycin D. (**C**) Relative expression of circHECA in SHF bulge of cashmere goats at telogen and anagen. (**D**) Efficiency evaluation of circHECA overexpression in SHF stem cells. (**E**) Overexpression of circHECA significantly upregulated expression of several indicator genes: KRT5, KRT8, KRT14, and KRT17 in SHF stem cells. (**F**) Overexpression of circHECA had no significant effect on linear HECA mRNA in SHF stem cells. ‘*’ and ‘**’ indicate significant difference at *p* < 0.05 and 0.01, respectively. ‘ns’ = no significant difference.

The circHECA was initially identified from the skin tissue of cashmere goats through amplifying divergent primers [27] with its potential regulatory network examined based on silico analysis [20]. Here, to further confirm that circHECA was truly circular, not a linear, trans-splicing, or genomic rearrangement product, total RNA from SHF stem cells was treated by RNase R. Subsequently, we evaluated the abundance of circHECA and linear HECA, where linear GAPDH mRNA was also detected as the internal reference by RT-qPCR. As a result, both linear HECA and GAPDH could be digested by RNase R, while circHECA was not affected by RNase R treatment (Figure 1A). On the other hand, we evaluated the degradation rate of circHECA through introducing actinomycin D, which intercepts the transcription of linear RNAs and circRNAs in SHF stem cells of cashmere goats. The obtained data showed that circHECA exhibited significantly slower degradation rates than linear HECA and GAPDH (Figure 1B), which suggested that circHECA had higher stability than linear HECA and GAPDH in SHF stem cells. These results strongly verify circHECA as a circular RNA molecule. Also, our data further support the previous findings that circRNAs are more resistant to RNase R degradation and have longer half-lives than linear RNA due to their covalently closed structures without 5′-3′ polarities and a polyadenylated tail [28].

It is widely accepted that the expression of circRNAs is strictly spatio-temporally specific across cell types, organs and tissues. We investigated the expression pattern of circHECA in the SHF bulge (the main nest of SHF stem cells) of cashmere goats at two key stages of the SHF cycle: telogen and anagen. As shown in Figure 1C, the expression of circHECA was significantly higher during anagen SHF bulge than during telogen (Figure 1C), which is supported by the data obtained from cashmere goat SHFs in a previous publication [20] and further implies its functional significance during the anagen stage of SHF stem cells of cashmere goats. Our current comparison focused on the functionally opposing telogen and anagen stages to specifically address circHECA’s role in SHF hair follicle activation in cashmere goats. The significant differences observed between the two key phases sufficiently support our core functional inference.

The knockdown analysis of circHECA implicated circHECA in the differentiation of SHF stem cells into hair follicle lineages [21]. Here, we overexpressed circHECA in SHF stem cells in cashmere goats (Figure 1D) with subsequent co-culture with dermal papilla cells for inducing differentiation into hair follicle lineages. The obtained results showed that the overexpression of circHECA could significantly enhance the mRNA expression of several indicator genes (of the differentiation of hair follicle stem cells into hair follicle lineages) including KRT5, KRT8, KRT14, and KRT17 (Figure 1E). The overexpression of circHECA did not affect the mRNA expression level of its host gene (HECA) in SHF stem cells (Figure 1F), which suggests that the HECA gene is not involved in the regulatory effect of circHECA on the differentiation of SHF stem cells into hair follicle lineages in cashmere goats.

### 3.2. Deficiency of m^6^A Modification Within circHECA Impedes Differentiation of SHF Stem Cells into Hair Follicle Lineages in Cashmere Goats

**Figure 2:**
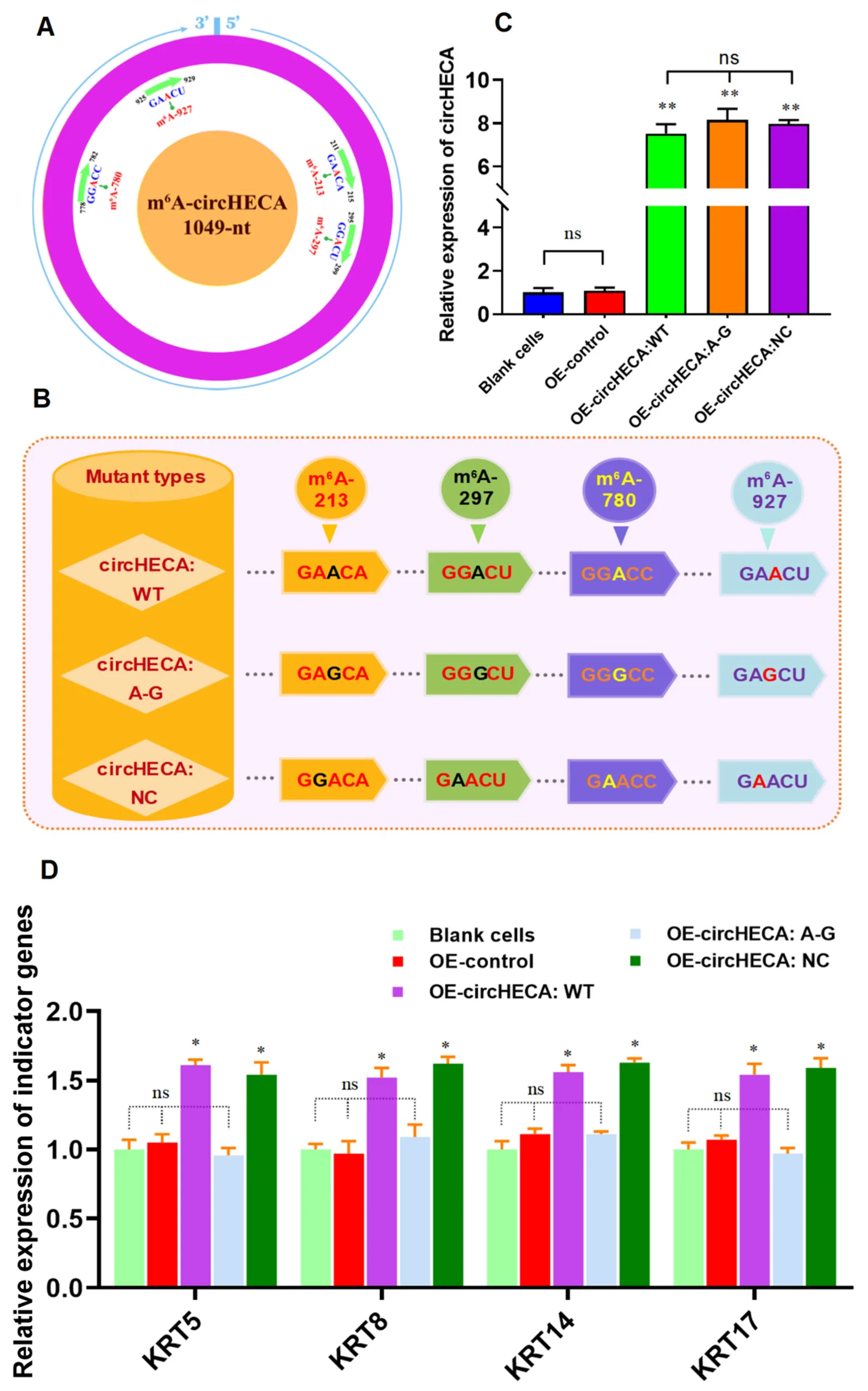
Deficiency of m^6^A modification within circHECA impedes differentiation of SHF stem cells into hair follicle lineages in cashmere goats. (**A**) Diagram of m^6^A site locations within circHECA molecule, with their corresponding motif structures. (**B**) Strategies for generating circHECA mutants against m^6^A modification sites. circHECA:WT = wild type of circHECA, circHECA: A-G = mutant of circHECA against m^6^A sites, and circHECA:NC = negative control mutant of circHECA against m^6^A sites. (**C**) Effects of m^6^A site mutation on circHECA expression in SHF stem cells. (**D**) Effects of m^6^A site mutation on expression of marker genes in SHF stem cells. Compared with OE-control, ‘*’ and ‘**’ indicate significant differences t *p* < 0.05 and 0.01, respectively. ‘ns’ = no significant difference.

In our previous investigation, as shown in Figure 2A, four m^6^A-modified sites were verified within the circHECA molecule through m^6^A-specific methylated RNA immunoprecipitation (Me-RIP) along with qPCR analysis, including m^6^A-213, m^6^A-297, m^6^A-780 and m^6^A-927 [20]. Interestingly, increasing evidence is significantly implicating m^6^A modifications within circRNA molecules in their functional effects [18,29]. Thus, to further confirm the significance of m^6^A modification in the functional effects of circHECA molecules, we constructed the circHECA overexpression vector (circHECA: WT), its mutation vector (circHECA m^6^A-deficient mutant, circHECA: A-G), and negative control mutant vector (circHECA m^6^A-decorated mutant, circHECA: NC) following the strategy scheme shown in Figure 2B. Subsequently, we transfected the constructed vectors (circHECA: WT, circHECA: A-G, and circHECA: NC) into SHF stem cells of cashmere goats to evaluate the significance of m^6^A modification in the functional effects of circHECA through inducing differentiation in in vitro assays. The overexpression of circHECA with different types of constructed vectors was confirmed through measuring their expression abundance in the transfected SHF stem cells (Figure 2C).

Meanwhile, we also noted that the introduction of mutations did not significantly change the expression level of circHECA in SHF stem cells (Figure 2C). Further, we assessed the expression changes in the indicator genes (in the differentiation of hair follicle stem cells) in the transfected cell lines with different types of constructed vectors including KRT5, KRT8, KRT14, and KRT17. As shown in Figure 2D, the ‘circHECA: WT’ cell lines, not the ‘circHECA: A-G’ cell lines, exhibited significantly higher expressions of the indicator genes compared with the ‘control’ cell lines (Figure 2D). Here, it can be excluded that the expression changes in the indicator genes between ‘circHECA: WT’ and ‘circHECA: A-G’ cell lines were caused by the expression abundance of circHECA in the corresponding cell lines, as we did not observed significant difference in expression abundance of circHECA between ‘circHECA: WT’ and ‘circHECA: A-G’ cell lines (Figure 2C). Interestingly, the transfection of the m^6^A-decorated mutant vector (circHECA: NC) rescued the reduced expression of the analyzed indicator genes in ‘circHECA: A-G’ transfected cell lines (circHECA m^6^A-deficience, Figure 2D). Based on the above results, it can be inferred that the deficiency of m^6^A modification within circHECA impeded the differentiation of SHF stem cells into hair follicle lineages in cashmere goats.

In other words, m^6^A modification is required for the promoting effect of circHECA on the differentiation of SHF stem cells into hair follicle lineages in cashmere goats. Such regulation of m^6^A modification within non-coding RNAs was also reported in several previous investigations, such as the m^6^A modification of long non-coding RNA: linc1281 in embryonic stem cells in mice [30], the m^6^A modification of circRNAs: circ-YAP in colorectal cancer cells [31], and both circRNA-ZFN638 [18] and circERCC6 [19] in SHF stem cells of goats.

### 3.3. CircHECA Recruits FUS Without Changing Its Expression in SHF Stem Cells in Cashmere Goats

**Figure 3:**
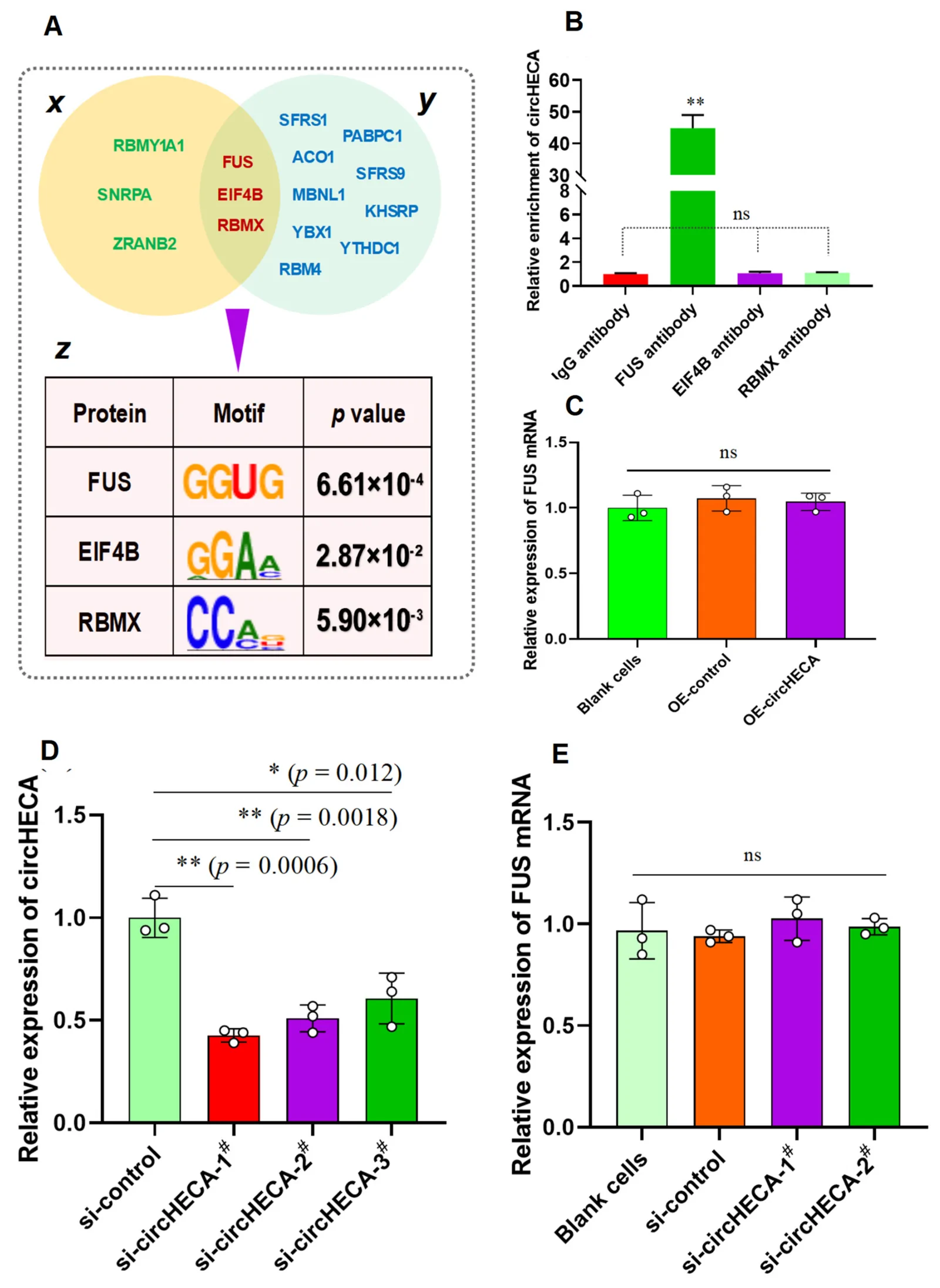
circHECA recruits FUS without changing its expression in SHF stem cells of cashmere goats. (**A**) Screening strategies of potential binding proteins of circHECA molecules. (**x**) Six RBPs having potential interaction relationships with circHECA molecules based on a previous publication [20]. (**y**) Twelve potential RBPs of circHECA molecules predicted using the RBPDB database (http://rbpdb.ccbr.utoronto.ca/, accessed on 12 August 2025). (**z**) Three potential RBPs of circHECA molecules picked out through taking an intersection from the above two groups: x and y with the corresponding motif structures and *p*-values. (**B**) Binding analysis of circHECA with FUS, EIF4B, and RBMX using RIP assays along with RT-qPCR assay. (**C**) Effect of circHECA overexpression on expression of FUS mRNA in SHF stem cells. (**D**) Knockdown efficiency of si-circHECA-1^#^, si-circHECA-2^#^, and si-circHECA-3^#^ for circHECA in SHF stem cells. (**E**) Effect of circHECA knockdown on expression of FUS mRNA in SHF stem cells. ‘*’ and ‘**’ indicate significant differences at *p* < 0.05 and 0.01, respectively. ‘ns’ = no significant difference.

Previous investigations demonstrated that circRNAs could function in biological cells via binding with RNA-binding proteins (RBPs) [32]. Moreover, circHECA was found to be mostly distributed in the cytoplasm of SHF stem cells in cashmere goats [20]. Thus, we hypothesized that circHECA positively regulates the differentiation of SHF stem cells into hair follicle lineages through interacting with RBPs. Six RBPs have potential interaction relationships with circHECA molecules including RBMY1A1, SNRPA, ZRANB2, FUS, EIF4B, and RBMX (Figure 3(Ax)) [20]. On the other hand, twelve potential RBPs of circHECA were predicted using the database of RNA-binding protein specificities, RBPDB (http://rbpdb.ccbr.utoronto.ca/, accessed on 12 August 2025) with a threshold value of 0.99 (Figure 3(Ay)). Thus, through taking an intersection from the above two groups (Figure 3(Ax),(Ay)), three potential RBPs of circHECA were picked out, including FUS, EIF4B, and RBMX, with the corresponding binding motif and *p*-value (Figure 3(Az)), where the respective *p*-value was further calculated through the RBP map (http://rbpmap.technion.ac.il/, accessed on 12 August 2025). 

To validate the binding of circHECA and the above three RBPs (FUS, EIF4B, and RBMX), we performed RIP assays along with RT-qPCR analysis. As a result, we found that circHECA largely bound to FUS but not EIF4B or RBMX (Figure 3B). To test whether circHECA can regulate FUS expression, we overexpressed circHECA in SHF stem cells through transfecting its overexpression vector. As shown in Figure 3C, circHECA had no significant effect on the FUS expression in the SHF stem cells of cashmere goats, which was further verified through the knockdown assay of circHCEA in SHF stem cells in cashmere goats with si-circHECA-1^#^ and 2^#^ (Figure 3D,E). These results suggest that circHECA recruit FUS but have no effect on its expression in SHF stem cells in cashmere goats. A highly similar functional mode was also reported in intrahepatic cholangiocarcinoma progression, where the circACTN4 was verified to recruit YBX1 but did not affect YBX1 mRNA expression in cholangiocarcinoma cell lines [22].

### 3.4. CircHECA Recruits FUS to Interact FOXM1 mRNA with Enhancing Its Stability in SHF Stem Cells in Cashmere Goats

**Figure 4:**
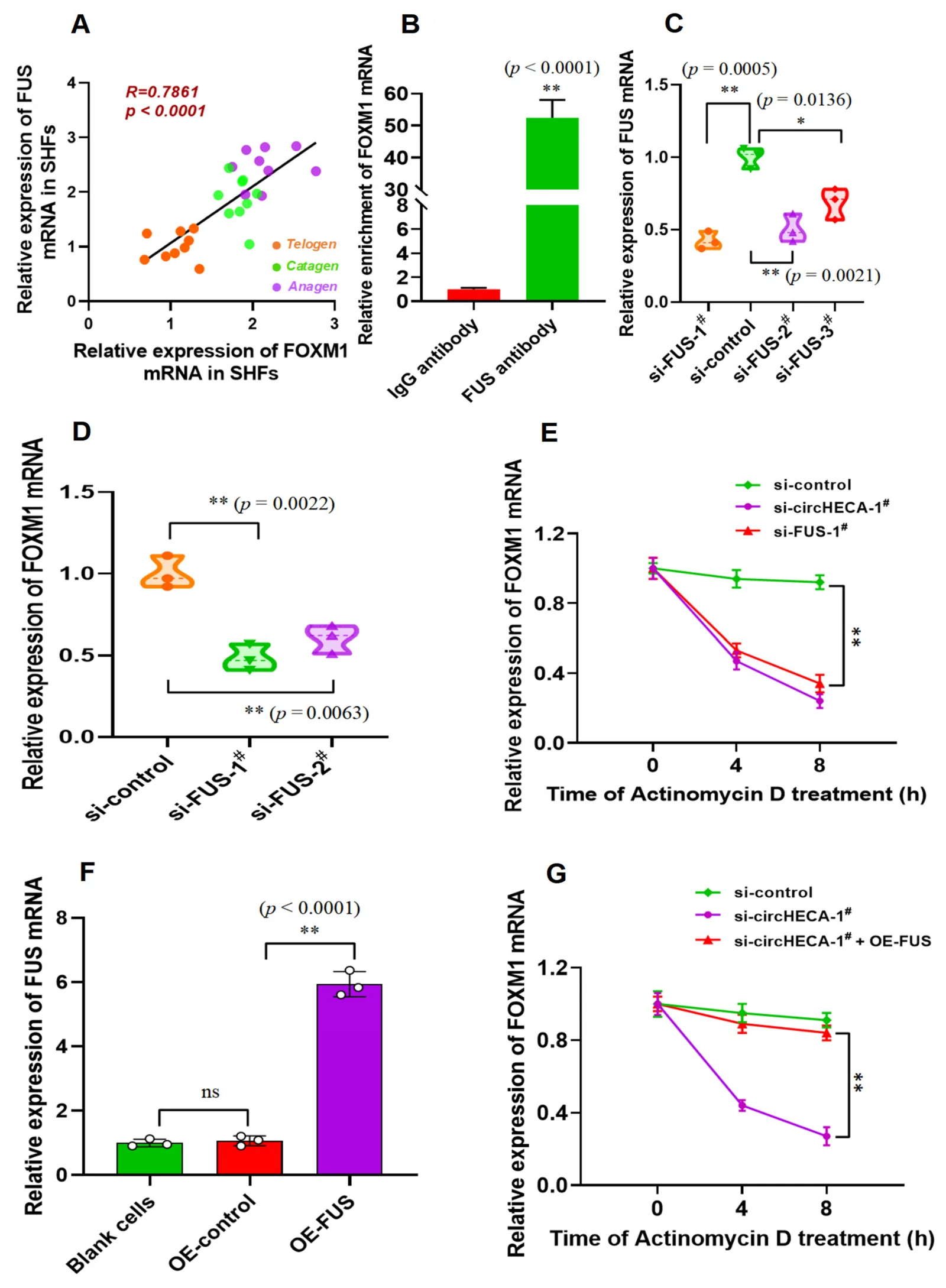
CircHECA recruits FUS to interact with FOXM1 mRNA, enhancing its stability in SHF stem cells in cashmere goats. (**A**) Expression correlation of FUS mRNA and FOXM1 mRNA in cashmere goat SHFs during SHF cycle: telogen, anagen and catagen, where scatter plot and fitted regression line are based on Pearson’s correlation. (**B**) Binding analysis of FUS with FOXM1 mRNA using RIP assays along with RT-qPCR assay. (**C**) Knockdown efficiency of si-FUS-1^#^, si-FUS-2^#^, and si-FUS-3^#^ to FUS in SHF stem cells. (**D**) Effect of FUS knockdown on expression of FOXM1 mRNA in SHF stem cells. (**E**) Stability analysis of FOXM1 mRNA in SHF stem cells with knockdown of circHECA or FUS upon treatment with actinomycin D. (**F**) Efficiency evaluation of FUS overexpression in SHF stem cells. (**G**) Stability of FOXM1 mRNA was evaluated in SHF stem cells transfected with si-control, si-circHECA-1^#^ and si-circHECA-1^#^ + OE-FUS upon treatment with actinomycin D. ‘*’ and ‘**’ indicate significant differences at *p* < 0.05 and 0.01, respectively. ‘ns’ = no significant difference.

Based on a previous study, the FUS, as an RNA-binding protein, binds to FOXM1 mRNA in the progression of invasive mucinous adenocarcinomas in the lungs in humans [33]. FOXM1, a member of the Forkhead box transcription factor family, is significantly involved in physiological process of various stem cells, such as neural stem cells [34] and glioma stem cells [35]. Moreover, although the biological functions of FOXM1 in SHF stem cells of cashmere goats still need to be further investigated, FOXM1 was also implicated in the induced activation of SHF stem cells in cashmere goats [25]. Thus, we have reason to ask whether FUS also binds to FOXM1 mRNA in SHF stem cells in cashmere goats. We firstly performed an expression correlation analysis between FUS and FOXM1 in SHFs of cashmere goats. Shown in Figure 4A, a significantly positive correlation was revealed in expression abundance between FUS and FOXM1 mRNAs in cashmere goat SHFs. Further, we performed RIP assays along with RT-qPCR analysis. The obtained results verified that FUS bound to FOXM1 mRNA in SHF stem cells in cashmere goats (Figure 4B).

To test whether FUS can regulate the expression level of FOXM1 mRNA in SHF stem cells in cashmere goats, we knocked down FUS expression through transfecting si-FUS-1^#^, -2^#^ and -3^#^ (Figure 4C). As shown in Figure 4D, the knockdown of FUS led to a significant decrease in FOXM1 mRNA abundance in SHF stem cells in cashmere goats (Figure 4D), which suggests that FUS is capable of positively regulating FOXM1 expression in SHF stem cells in cashmere goats. These results are highly similar to those reported in human cardiac hypertrophy where FUS was found to bind with TLR4 mRNA and to positively regulate its expression in human cardiomyocytes [24]. On the other hand, we also noted that the stability of FOXM1 mRNA declined when circHECA or FUS was knocked down in SHF stem cells in cashmere goats (Figure 4E). Interestingly, we found that the reduced stability of FOXM1 mRNA caused by the knockdown of circHECA was rescued by the overexpression of FUS through co-transfecting with pcDNA3.1-FUS (Figure 4F,G). Taken together with our above results, it can be inferred that circHECA recruits FUS to interact with FOXM1 mRNA, enhancing its stability in SHF stem cells of cashmere goats.

It is widely accepted that circRNAs bind to RNA binding proteins to regulate gene expression at the post-transcriptional level [36]. FUS is known as an RNA-binding RNA that stabilizes mRNA in the cytoplasm [37]. In human cardiomyocytes, it was reported that circTLR4 promotes cardiac hypertrophy through recruiting FUS to stabilize TLR4 mRNA [24], which is similar to our result that circHECA recruits FUS to bind with FOXM1 mRNA (Figure 4B), thereby enhancing its stabilization in SHF stem cells in cashmere goats (Figure 4E,G). This regulatory mode was also reported in the progression of invasive mucinous adenocarcinomas (IMA) of the lung in humans, where HOXC-AS3 (a long non-coding RNA) recruited FUS to stabilize FOXM1 mRNA, thereby accelerating the progression of invasive mucinous adenocarcinoma in the lung [33].

### 3.5. CircHECA Relying on m^6^A Modification to Activate NOTCH Signaling Pathway Through FUS/FOXM1 Axis

**Figure 5:**
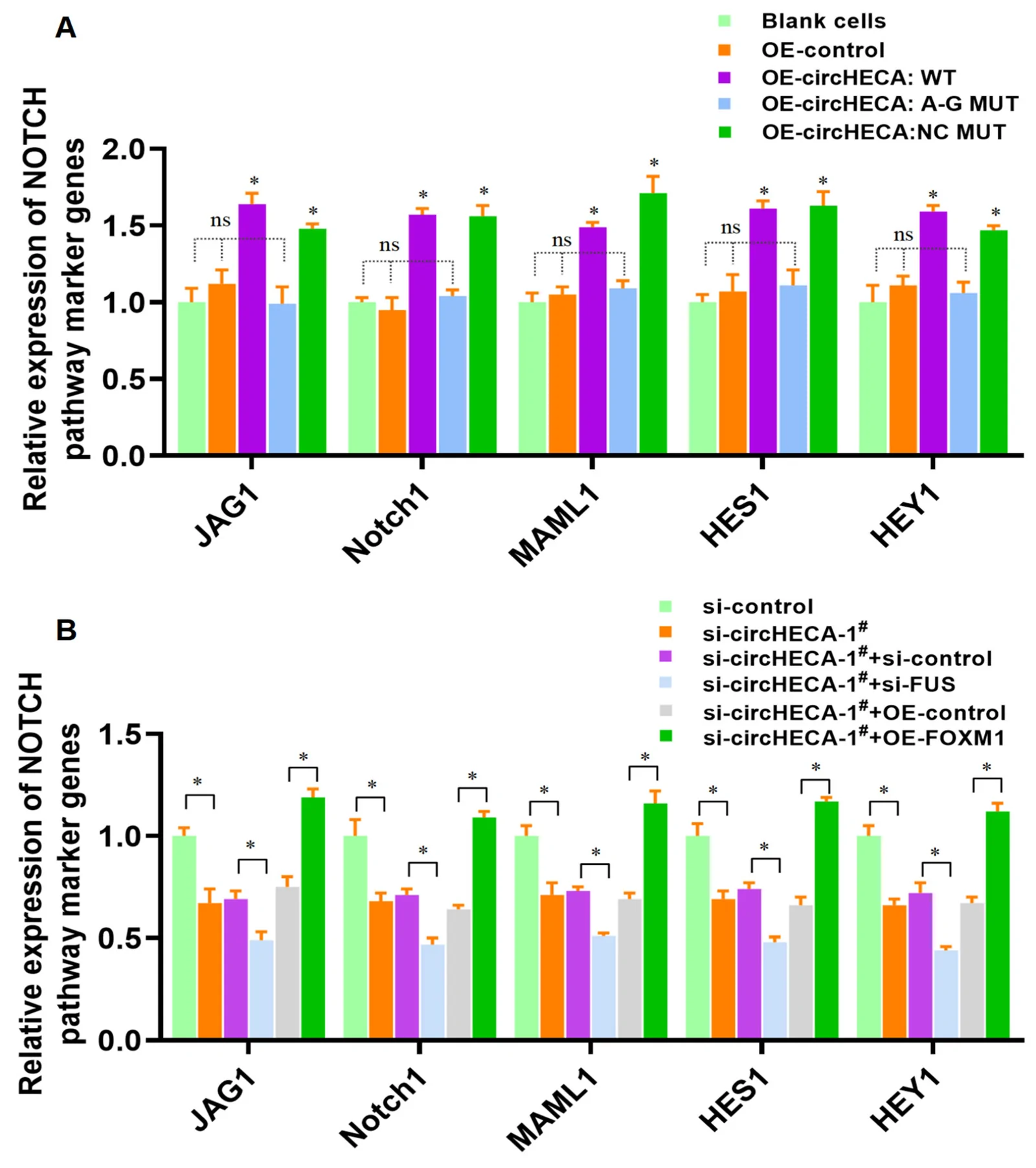
circHECA relies on m^6^A modification to activate NOTCH signaling pathway via FUS/FOXM1 axis. (**A**) Effects of m^6^A site mutation in circHECA molecule on expression of NOTCH signaling pathway-related genes in SHF stem cells. ‘*’ indicates significant difference compared with OE-control, *p* < 0.05. ‘ns’ = no significant difference. (**B**) circHECA activates NOTCH signaling pathway via FUS/FOXM1 axis. ‘*’ indicates significant difference between two analyzed groups, *p* < 0.05.

Based on our above results, circHECA positively regulates the stabilization of FOXM1 mRNA in SHF stem cells in cashmere goats through recruiting FUS protein. There is evidence that FOXM1 can activate the NOTCH signaling pathway [38], which may be capable of promoting the differentiation of hair follicle stem cells into hair follicle lineages in cashmere goats [39]. This drove us to ask whether circHECA relies on m^6^A modification to activate the NOTCH pathway through the FUS/FOXM1 axis in SHF stem cells in cashmere goats. To explore this hypothesis, we further analyzed the effect of circHECA in relying on m^6^A modification on the NOTCH signaling pathway via the FUS/FOXM1 axis, which was assessed by investigating marker molecules of NOTCH pathway including JAG1, NOTCH1, MAML1, HES1, and HEY1. As shown in Figure 5A, compared with the ‘OE-control’ cell lines, the ‘OE-circHECA: WT’ cell lines exhibited significantly higher mRNA expression of the analyzed marker genes, but not the ’circHECA: A-G’ cell lines. The ‘circHECA: NC’ cell lines (m^6^A-decorated mutant) rescued the reduced mRNA expression of the marker genes in circHECA: A-G’ cell lines (m^6^A-deficient mutants, Figure 5A). These results suggest that circHECA activates the NOTCH signaling pathway, for which m^6^A modifications are necessary.

In this case, we further asked whether the FUS/FOXM1 axis is responsible for the effect of m^6^A-circHECA on activating the NOTCH signaling pathway in SHF stem cells in cashmere goats. To verify this hypothesis, we knocked down FUS or overexpressed FOXM1 in SHF stem cells with circHECA knockdown. As shown in Figure 5B, the knockdown of circHECA (si-circHECA-1^#^) led to significant decreases in mRNA expressions of NOTCH signaling pathway marker genes (JAG1, NOTCH1, MAML1, HES1, and HEY1) compared with the control cell lines (si-control). Interestingly, these observed effects were further exacerbated through the transfection of si-FUS (si-circHECA-1^#^ + si-FUS) but were counterbalanced through the introduction of OE-FOXM1 (si-circHECA-1^#^ + OE-FOXM1) in SHF stem cells in cashmere goats (Figure 5B). Taken together, circHECA relies on m^6^A modification to activate the NOTCH pathway through the FUS/FOXM1 axis.

**Figure 6:**
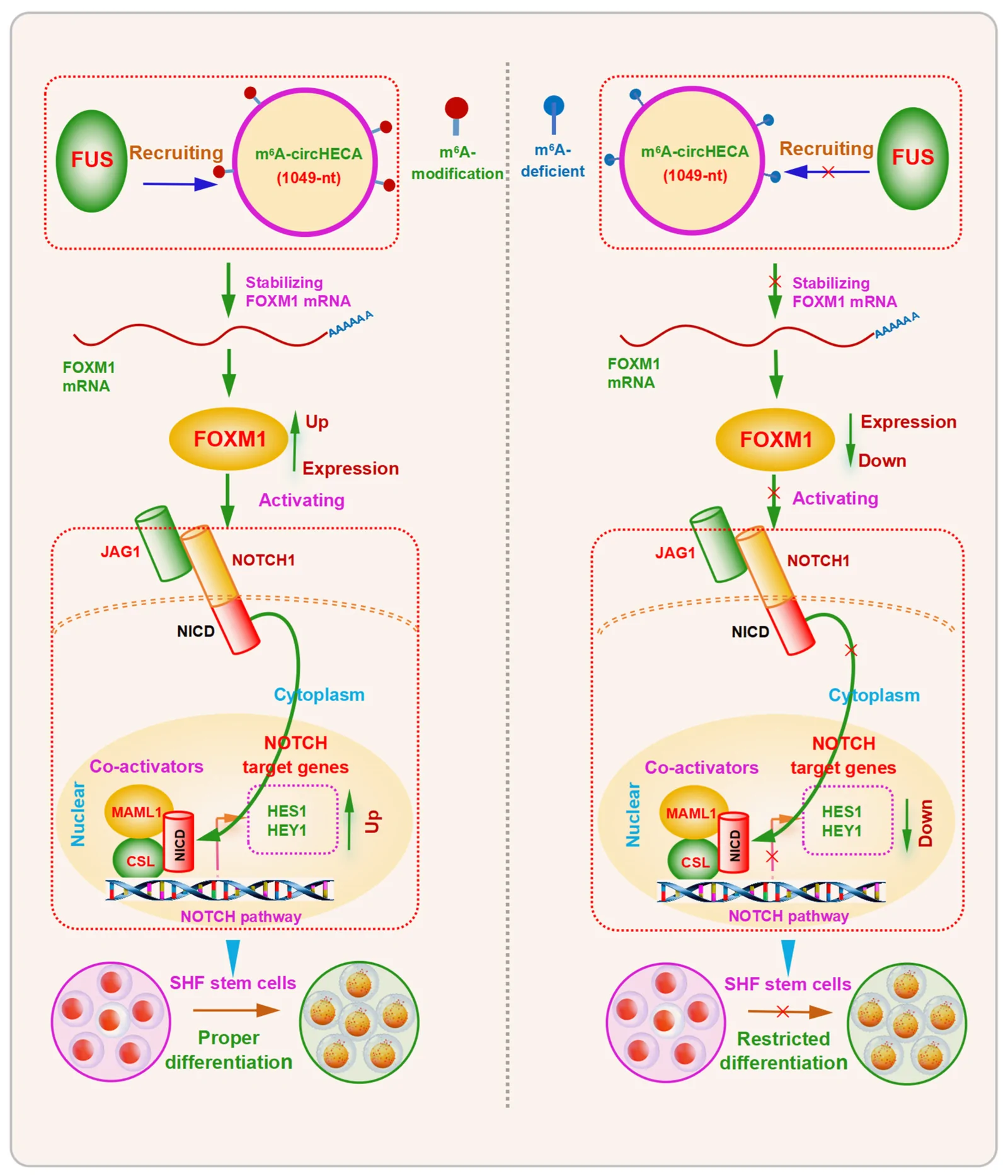
Schematic representation of m^6^A-circHECA recruiting FUS promoting differentiation of SHF stem cells into hair follicle lineages through stabilizing FOXM1 mRNA to activate NOTCH pathway in cashmere goats.

As well-known, SHF stem cells are responsible for SHF development and regeneration in cashmere goats [9]. NOTCH signaling is heavily implicated in the self-renewal, proliferation, and differentiation of hair follicle stem cells [40]. Previous studies showed that intact NOTCH signaling is required in the postnatal development of hair follicles; moreover, NOTCH was thought to control the differentiation fate switch of hair follicle stem cells in the bulge (the main nest of hair follicle stem cells), preventing them from adopting an epidermal fate [41]. Also, there is evidence that the activation of the NOTCH signaling pathway accelerates the differentiation of hair follicle stem cells [39]. In this study, m^6^A-circHECA was identified as a positive activator of the NOTCH pathway in cashmere goats. Although it is not yet known whether there is other regulatory ways by which m^6^A-circHECA is involved in activating the NOTCH pathway, we showed that m^6^A-circHECA positively regulated the NOTCH signaling pathway by recruiting FUS to stabilize FOXM1 mRNA in SHF stem cells in cashmere goats. Thus, we revealed a new regulatory pathway: m^6^A-circHECA recruiting FUS promotes the differentiation of SHF stem cells into hair follicle lineages through stabilizing FOXM1 mRNA to activate the NOTCH pathway in cashmere goats (Figure 6).

However, we also recognize a limitation of this study: due to the lack of specific antibodies for goats, we were unable to validate the expression changes in the implicated genes at the protein level. Nevertheless, our conclusions are robustly supported by the consistent and directional mRNA expression changes observed. We sincerely hope that future studies, potentially through custom generation of goat-specific antibodies or alternative antibody-independent proteomic approaches, will further verify the functional significance of m6A-circHECA in cashmere goat SHF stem cells.

## 4. Conclusions

Here, we demonstrated that m^6^A-circHECA recruiting FUS promotes the differentiation of SHF stem cells into hair follicle lineages in vitro through stabilizing FOXM1 mRNA to activate the NOTCH pathway in cashmere goats. These findings provide a potential target for regulating the differentiation of SHF stem cells into hair follicle lineages in cashmere goats.

## Figures and Tables

**Table 1:** The information on the PCR primers that were used in this investigation.

Genes	References	Primer Pair with Sequence (5′–3′) ^a^	Primer Length (nt)	Annealing Temperature (°C)	Amplicon Size (bp)
circHECA	Shen et al., 2024 [20]	F:ACGATGTTCCCTGTCACCTT	20	57	87
(divergent primers)		R: TCGTCTTTCTCCAGGTCCAC	20		
HECA	XM_018053273.1 in GenBank ^b^	F:CGGGGTTGTCGGTTCATAGAG	21	56	117
		R: CGTGGAAAGTGTTCAGCTTGT	21		
GAPDH	Yin et al., 2023 [18]	F: TGAACCACGAGAAGTATAACAACA	24	53	125
		R: GGTCATAAGTCCCTCCACGAT	21		
KRT5	XM_018047718.1 in GenBank	F:AGGTCTAGAGGATAAAAGCG	20	54	126
		R: AGTGGAGTAAGAGTAGCGGG	20		
KRT8	Yin et al., 2020 [7]	F: TCCTTCAGCAGCCGCTCCTA	20	58	160
		R: CTGTAATGCCCCCCAAACCT	20		
KRT14	XM_005693818.3 in GenBank	F:GCTATGGTGTTGGTGATGGG	20	56	198
		R:TGGTCTTGAAGTAGGGGCTG	20		
KRT17	Yin et al., 2020 [7]	F: GGGGAATGGAAACAGAGGAG	20	56	112
		R: GAGGAGAGAAGCCCAAGATG	20		
FUS	XM_018040751.1 in GenBank	F:TAAGAAAACAGGACAGCCCA	20	53	126
		R:CCATCAAACCAGTCAATAGC	20		
FOXM1	Xu et al., 2024 [25]	F:CAAGAGGTGGAAGAAAAGGAGA	22	56	140
		R:ATCAGGGCCATGTAGGAGTATG	22		
JAG1	XM_018056924.1 in GenBank	F:ATGCGTGGATGGTGAGAACTG	21	56	105
		R:AAGGCACAAGGGGAAGACTGG	21		
NOTCH1	XM_018056124.1 in GenBank	F:CAAGGCTCGGAGGAAGAA	18	57	156
		R:GAGGGAGACGGCTGGAAA	18		
MAML1	XM_018051602.1 in GenBank	F:ACCCTCCCAATGTCCCTG	18	55	172
		R:TGCTGCTCCCTCTGCTTC			
HES1	XM_018046555.1 in GenBank	F:GCCAGTTTGCCTTCCTCA	18	56	162
		R:AGTTCCGCCACGGTCTCC	18		
HEY1	XM_018058382.1 in GenBank	F:GCTCCTTTCACTTACTGTCTCC	22	53	144
		R:GCTTTCCCCTCCCTCATTCTAC	22		

^a^ F: forward, R: reverse. ^b^ GenBank: https://www.ncbi.nlm.nih.gov (accessed on 9 March 2025).

## Data Availability

The data presented in this study are not publicly available and are available on request from the corresponding author.

## Abbreviations

The following abbreviations are used in this manuscript:

m^6^A	*N*^6^-methyladenosine
circRNA	Circular RNA
SHF	Secondary hair follicle
Me-RIP	m^6^A-specific methylated RNA immunoprecipitation
RIP	RNA immunoprecipitation
RBP	RNA-binding protein
RT-qPCR	Reverse-transcription quantitative polymerase chain reaction
DPC	Dermal papilla cell
